# CRY-dependent plasticity of tetrad presynaptic sites in the visual system of *Drosophila* at the morning peak of activity and sleep

**DOI:** 10.1038/s41598-020-74442-w

**Published:** 2020-10-23

**Authors:** Milena Damulewicz, Olga Woźnicka, Małgorzata Jasińska, Elżbieta Pyza

**Affiliations:** 1 Department of Cell Biology and Imaging, Institute of Zoology and Biomedical Research, Jagiellonian University, Kraków, Poland; 2 Department of Histology, Jagiellonian University Medical College, Kraków, Poland

**Keywords:** Cell biology, Neuroscience

## Abstract

Tetrad synapses are formed between the retina photoreceptor terminals and postsynaptic cells in the first optic neuropil (lamina) of *Drosophila.* They are remodelled in the course of the day and show distinct functional changes during activity and sleep. These changes result from fast degradation of the presynaptic scaffolding protein Bruchpilot (BRP) by Cryptochrome (CRY) in the morning and depend on BRP-170, one of two BRP isoforms. This process also affects the number of synaptic vesicles, both clear and dense-core, delivered to the presynaptic elements. In *cry*^01^ mutants lacking CRY and in *brp*^Δ170^, the number of synaptic vesicles is lower in the morning peak of activity than during night-sleep while in wild-type flies the number of synaptic vesicles is similar at these two time points. CRY may also set phase of the circadian rhythm in plasticity of synapses. The process of synapse remodelling stimulates the formation of clear synaptic vesicles in the morning. They carry histamine, a neurotransmitter in tetrad synapses and seem to be formed from glial capitate projections inside the photoreceptor terminals. In turn dense-core vesicles probably carry synaptic proteins building the tetrad presynaptic element.

## Introduction

Circadian clocks generate circadian rhythms in various processes, including neuronal and synaptic plasticity in the brain. In the visual system of *Drosophila melanogaster* and in the somatosensory cortex of mice, there are circadian changes in the number and structure of synapses^1,2^.

The visual system of the fruit fly is a convenient model to study synaptic plasticity. It is composed of the retina and four optic neuropils: the lamina, medulla, lobula and lobula plate. In the lamina, the first optic neuropil, light signals are integrated, filtered, processed and sent to deeper parts of the brain. The most numerous synapses in the lamina are tetrad ones formed between the photoreceptor terminals and four lamina post-synaptic cells. Two of them, L1 and L2 monopolar cells, have been described, and circadian changes in their morphology are well documented. The L1 and L2 axons are larger in diameter twice a day: at the beginning of the day and at the beginning of the night in *Drosophila*; concomitantly, the axons also change their shape from conical to cylindrical. These changes are evident in light/dark (LD12:12) conditions and in constant darkness (DD), which means that the circadian clock controls their cyclic morphological plasticity^1,3^. Moreover, under constant light (LL), the size of L1 and L2 axons is larger, while in DD it is smaller relative to L1 and L2 axon sizes in LD12:12^4^. Circadian changes in the size of neurons are offset by changes in glial cells^5^, which surround lamina cartridges enclosing the photoreceptor terminals R1–R6, axons of R7, R8, lamina interneurons L1–L5, and processes of some other neurons, including those located outside the lamina. In addition to axons, the L2 nuclei and dendrites also show rhythmic changes in their size. Dendrites are longer at the beginning of the day when flies are highly active and shorter at night when they sleep^6^.

The tetrad synapses transmit photic and visual information using histamine as a neurotransmitter^7^, however, the inner photoreceptors (R8) which terminate in the medulla, also use acetylcholine as a neurotransmitter^8^. Another type of plastic synapses, feedback synapses, are formed between L2 and photoreceptor terminals^9^, and they seem to modulate the activity of photoreceptors during the rest time and increase their sensitivity during low light intensity at night^10,11^. It is possible that others or all types of synapses in the visual system show circadian plasticity, but this has not been studied yet.

The numbers of T-bars of tetrad synapses show rhythmic changes^12,13^, and this rhythm is correlated with the rhythm in L1 and L2 morphological changes. Both light and clock regulate the rhythm in tetrad synapse frequency because the morning peak disappears under DD and in clock mutants. Two peaks in the frequency of tetrad synapses in LD12:12 are also correlated with two, morning and evening, peaks in locomotor activity^14^. Moreover, several synaptic proteins oscillate in abundance during the day/night cycle and in constant darkness in the lamina^15,16^. It indicates that their levels are regulated by the circadian clock and light, however, locomotor stimulation affects the daily pattern of their expression.

All synapses in the nervous system of *Drosophila* and other insects possess a characteristic presynaptic element called the T-bar, and one of the most important proteins in the T-bar is Bruchpilot (BRP), a large coiled-coil domain protein with homology to a family of mammalian active zone proteins^17–20^. BRP is expressed in two isoforms, 170 kDa and 190 kDa, which are involved in circadian synaptic plasticity^14^, but their specific functions are unknown. Mutants of BRP show defective active zone membranes within T-bars, a complete loss of presynaptic specializations and diminished release of neurotransmitters^21^. Thus, the synaptic activity is lower in *brp* mutants than in wild-type flies. Moreover, BRP is required for the formation of olfactory memory^22^, and abnormalities in the active zone have been observed in several *Drosophila* disease models. For example, density of the active zone was decreased in models of amyotrophic lateral sclerosis and Pitt-Hopkins syndrome^23,24^. BRP undergoes post-translational modifications and is acetylated by elongator complex protein 3 (ELP3) acetyltransferase and deacetylated by histone deacetylase 6 (HDAC6)^25,26^.

In the lamina BRP protein expression oscillates during the day with two maxima—at the beginning of the day and at the beginning of the night^15^. Rhythmic expression depends on the circadian clock, as it disappears in *per*^*0*^ mutants. Light also controls the morning peak in BRP expression; *norpA* mutants (lacking phospholipase C, a key factor in the phototransduction pathway) or wild-type flies under DD conditions both lack the peak at the beginning of the day. On the other hand, the evening peak of BRP expression depends on CRY and the clock protein TIMELESS (TIM)^15^. Moreover, *cry*^*0*^ mutants do not show decreases in BRP level at ZT4, suggesting that CRY is involved in BRP turnover during the day.

CRY is a photosensitive protein that changes its conformation after light exposure. In this form, it binds to TIM, triggering its ubiquitination and degradation. It has been shown that CRY plays a similar role for BRP^27^.

In addition to its clock function, CRY regulates neuronal activity. It accumulates in dendritic and axonal termini^28^ and confers light-dependent excitability even when ectopically expressed^29^. Interestingly, CRY controls the shape of the L2 dendrites, as they appear irregular in *cry*^*b*^ mutants^6^. In addition to TIM, CRY is able to form complexes with many different proteins including NinaC, InaD and RDGA, which are elements of the phototransduction pathway^30^.

The aim of this study was to analyse the ultrastructure of tetrad presynaptic elements in wild-type flies, *brp*^Δ170^, *brp*^Δ190^ and *cry*^*01*^ mutants collected at different times during the day, specifically at the morning peak of activity and during night-sleep, to learn how the ultrastructure of the tetrad synapse T-bar and the number of synaptic vesicles are modified in the course of the day and adapt to changes in light intensity and the fruit fly’s behaviour. Moreover, we examined the role of BRP isoforms and how CRY regulates the circadian plasticity of tetrad synapses.

High resolution and tomography TEM studies (EMT) of synapses are rare, and in *Drosophila* they have been carried out mostly on the neuromuscular junctions (NMJ). Our present study is the first to show cyclic remodelling of the presynaptic sites in the fly’s brain at the EM level and involvement of the CRY protein in this process.

## Results

### Ultrastructure of tetrad presynaptic profiles in Canton S, ***brp***^Δ170^, ***brp***^Δ190^ and ***cry***^***01***^ mutants in LD 12:12

**Figure 1:**
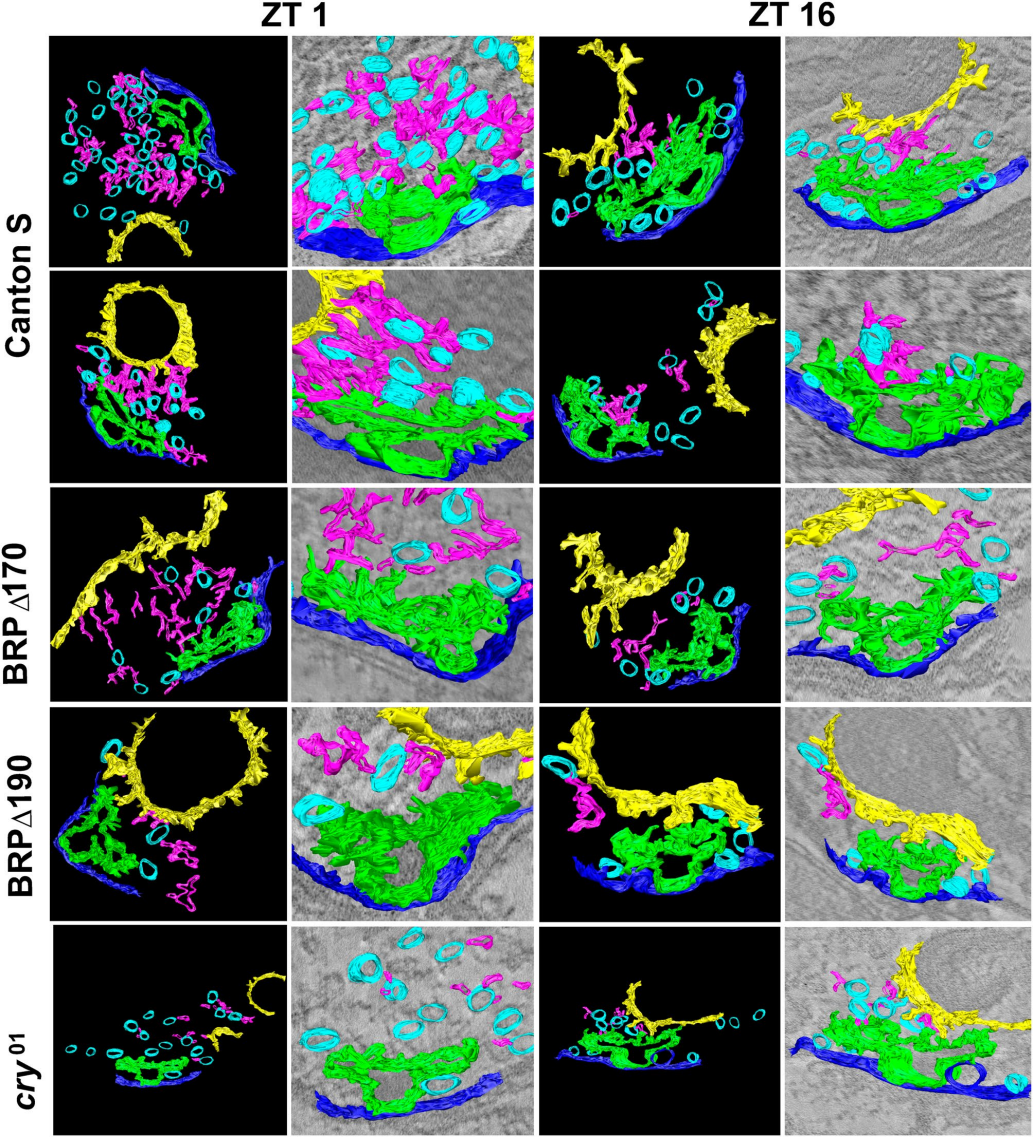
Electron microscope tomography (EMT) 3D reconstructions of tetrad presynaptic sites in Canton S and mutants *brp*^Δ170^, *brp*^Δ190^ and *cry*^01^ at ZT1 (morning peak of activity) and ZT16 (sleep). Left panels in ZT1 and ZT16 columns show reconstructions of the selected structures: T-bars (green), synaptic vesicles (blue), filamentous proteins attached to vesicles (pink), capitate projections (yellow). Right panels in ZT1 and ZT16 columns show original micrographs obtained from EMT.

The ultrastructure of tetrad presynaptic elements in Canton S, examined with EMT, was different at ZT1 (activity) when compared with that at ZT16 (sleep). At ZT1, more synaptic vesicles were attached to the T-bar platform and to capitate projections with filamentous proteins than at ZT16 (Fig. 1, Figure S1). Capitate projections are invaginations from the epithelial glial cells to the photoreceptor terminals in the lamina. They oscillate in the number in *Drosophila* photoreceptors, with more shallow capitate projections during the day and deep ones during the night^31^. Vesicles attached to capitate projections were detected only during tomographic reconstructions and they were not visible on conventional TEM micrographs or reconstructions from 10–12 ultrathin (70 nm) sections. Tomographic reconstructions were only used to visualise the ultrastructure of T-bars and elements near T-bars. Because of a large quantity of data obtained during EM tomography, 120 images from one 150 nm section, only the middle part of each synapse could be reconstructed.

The daily changes in the ultrastructure of the presynaptic sites were not observed in *brp*^Δ170^, *brp*^Δ190^ mutants (Fig. 1). In both mutants, there were fewer filamentous proteins and vesicles tethered to the T-bar than in Canton S. In addition, there were no differences in the number of synaptic vesicles tethered to T-bars at ZT1 and ZT16. In *brp*^Δ190^, the structure of the T-bar was less stable with fewer elements than in *brp*^Δ170^ and Canton S. In *cry*^01^ mutants, synaptic vesicles were not tethered to the T-bar platform during the day (ZT1), and there were more filamentous proteins and vesicles tethered to the T-bar and capitate projections during the night (ZT16, sleep) (Fig. 1).

**Figure 2:**
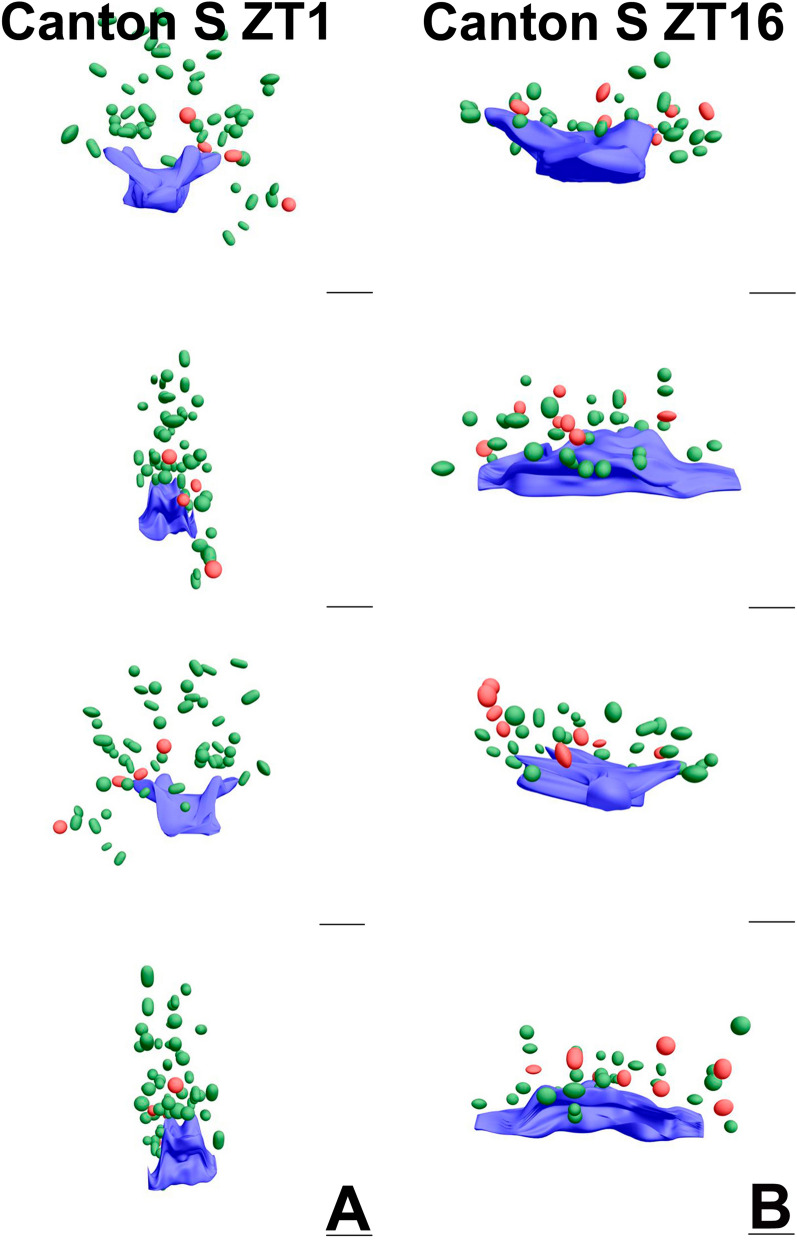
Four side views of TEM 3D reconstructions from serial sections of T-bars (blue) with synaptic vesicles (clear—green, dense-core—red) in Canton S at ZT1 and ZT16. Scale bar: 100 nm.

**Figure 3:**
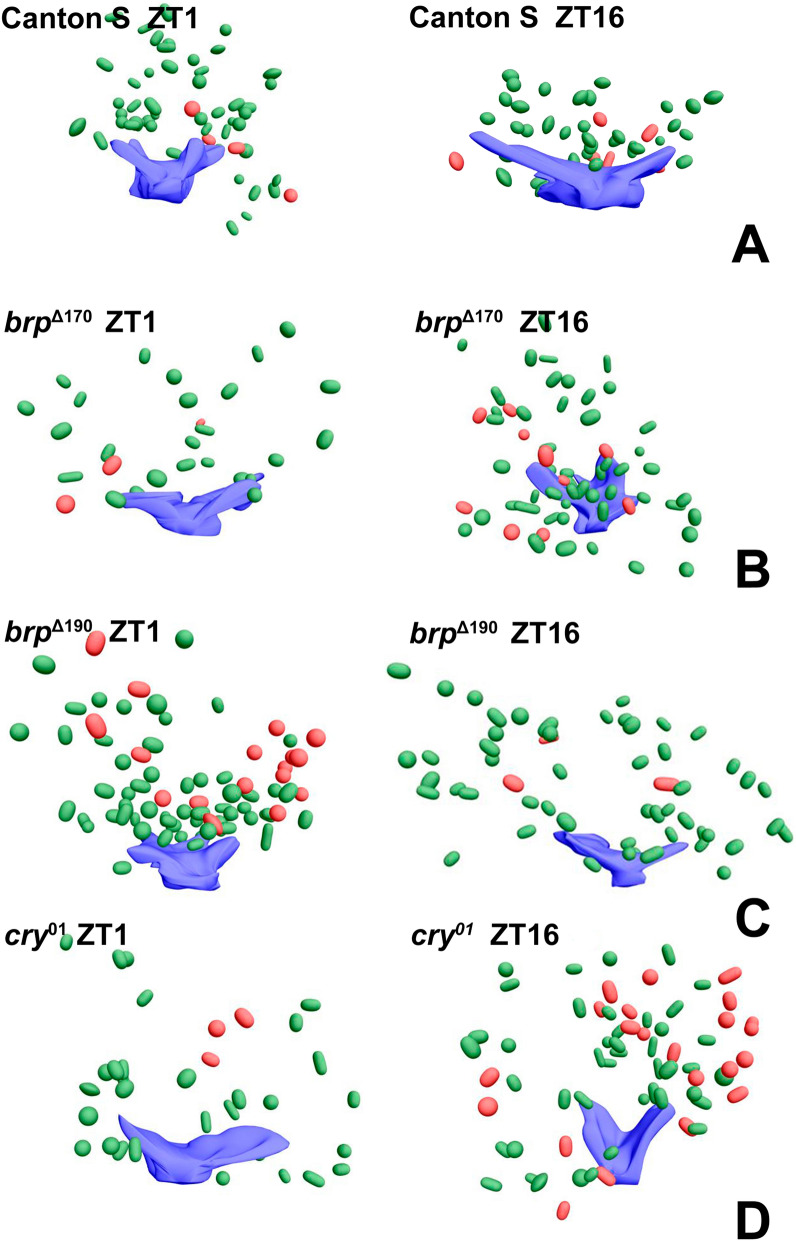
Largest view of TEM 3D reconstructions from serial sections of T-bars (blue) with synaptic vesicles (clear—green, dense-core—red) in Canton S, *brp*^Δ170^, *brp*^Δ190^ and *cry*^01^ at ZT1 and ZT16. Scale bar: 100 nm.

Moreover, using the EMT method, we identified two types of synaptic vesicles, clear and dense-core vesicles (Figs. 2, 3, S1–3). The dense-core vesicles were less numerous than clear ones. All synaptic vesicles in the photoreceptor terminals were distinguished from other membranous structures by their size of approximately 30–50 nm and their elongated shape. Vesicles attached to capitate projections were observed only during the day.

Reconstructions of T-bars and synaptic vesicles, both clear and dense-core, located next to T-bars and at a distance up to 400 nm from TEM serial sections showed larger T-bar platforms during the night than during the day in Canton S and similar numbers of vesicles at ZT1 and ZT16 (Figs. 2, 3A, S3A, S4). However, their numbers were different in mutants (Figures S3B-D, S5-7). In *brp*^Δ170^, more vesicles were observed at ZT16 than at ZT1, but in case of *brp*^Δ190^, this pattern was reversed, with more vesicles at ZT1 than at ZT16 (Figs. 3B–D, S3B, C, S5,6). The size of the platform was similar at both time points in the two mutants. In *cry*^01^, more vesicles were found at ZT16, but the T-bar platform was larger at ZT1 (Fig. 3D, S3D, S7).

### Distribution of synaptic vesicles and T-bar platform volume in tetrad presynaptic sites

**Figure 4:**
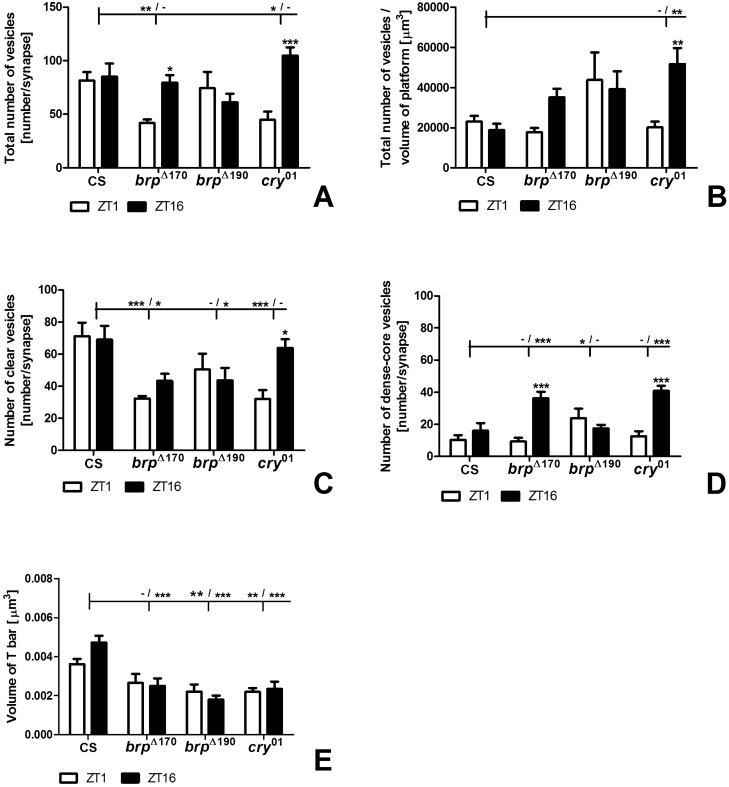
(**A**–**E**) Numbers of vesicles (total, clear, dense-core and per volume of platform) and size of the T-bar platform volume of tetrad synapses in Canton S, *brp*^Δ170^, *brp*^Δ190^ and *cry*^01^ at ZT1 and ZT16. Asterisks indicate the following statistically significant differences: *p < 0.05; **p < 0.01; ***p < 0.001. Asterisks above bars show statistically significant differences between ZT1 and ZT16 within every group. Asterisks above horizontal lines show statistically significant differences between Canton S and mutants (*brp*^Δ170^**,** *brp*^Δ190^ and *cry*^01^), and minus means the lack of a statistically significant difference. Means ± SEM.

The analysis of total synaptic vesicle number per synapse showed that in Canton S, the number of vesicles was similar at the morning peak of activity (ZT1) and in the middle of the night (ZT16) when flies sleep. In *brp*^Δ190^, the number was also similar at ZT1 and ZT16; however, in *brp*^Δ170^ and *cry*^01^ mutants, the number of vesicles was higher by approximately 90% and 2.34 times, respectively, during the night than in the morning (two-way ANOVA, *p* = 0.0017, F_57_ = 10.9; BRP Δ170: *p* < 0.05, t = 2.8; *cry*^01^: *p* < 0.001, t = 4.454) (Fig. 4). There were more vesicles, by about 95% and 82% in Canton S at ZT1 than in *brp*^Δ170^ (two way ANOVA, *p* = 0.0012, F_57_ = 6.068; *p* < 0.01, t = 2.939) and *cry*^01^ (*p* < 0.05, t = 2.717), respectively (Fig. 4A). When the number of all vesicles was analysed per 1 µm^3^ volume of the T-bar platform, there were no statistically significant differences between ZT1 and ZT16 except in *cry*^01^ (two way ANOVA, *p* = 0.0404, F_57_ = 4.398; *p* < 0.01, t = 3.258). A difference was also observed between Canton S and *cry*^01^ mutants at ZT16, with more vesicles in *cry*^01^ (two way ANOVA, *p* = 0.0166, F_57_ = 3.704; *p* < 0.01, t = 3.494) (Fig. 4B).

Among the two types of synaptic vesicles identified, the number of clear ones was higher than the number of dense-core vesicles. In Canton S wild-type flies and mutants, differences between ZT1 and ZT16 were not statistically significant except for *cry*^01^, which had about 99% more clear vesicles during the night (two-way ANOVA, *p* = 0.0394, F_57_ = 2.968; *p* < 0.05, t = 3.151) (Fig. 4C). There were also statistically significant differences between Canton S and *brp*^Δ170^ at ZT1, and ZT16 with 2.2 times and 60% more vesicles, respectively in Canton S (two-way ANOVA, *p* = 0.0003, F_57_ = 7.481; ZT1 *p* < 0.001, t = 3.845; ZT16 *p* < 0.05, t = 2.629), as well as between Canton S and *brp*^Δ190^ at ZT16 with 58% more vesicles in Canton S (*p* < 0.05, t = 2.590) and between Canton S and *cry*^01^ at ZT1 (*p* < 0.001, t = 3.870) (Fig. 4C) with 2.2 times more clear vesicles in Canton S. It means that CRY and BRP-190 are important for the recruitment of synaptic vesicles to the T-bar and their higher turnover at ZT1 than ZT16. In result a similar number of clear synaptic vesicles was detected during activity and sleep in wild-type flies. In contrast BRP-170 seems to be responsible for maintaining the proper number of vesicles at ZT16.

In the case of dense-core vesicles, their number was significantly higher (2.25 times) in *brp*^Δ170^ than in Canton S at ZT16 (two-way ANOVA, *p* < 0.0001, F_57_ = 9.744; *p* < 0.001, t = 3.816), in *brp*^Δ190^ than in Canton S (2.33 times) at ZT1 (*p* < 0.05, t = 2.510), and in *cry*^01^ than in Canton S (2.54 times) at ZT16 (*p* < 0.001, t = 4.694) (Fig. 4D). In contrast to the clear vesicles, the number of dense-core vesicles was increased at ZT1 in *brp*^Δ190^ but at ZT16 in *brp*^Δ170^ and *cry*^01^ in comparing with Canton S. The day/night difference in the number of dense-core synaptic vesicles was detected only in *brp*^Δ170^ (two-way ANOVA, *p* < 0.0001, F_57_ = 25.64; *p* < 0.001, t = 4.949 and *cry*^01^ (*p* < 0.001, t = 5.202), with more vesicles during the night, 3.87 and 3.24 times, respectively (Fig. 4D). BRP-170 and CRY are involved in decreasing the number of dense-core vesicles during the night.

In addition we also measured the T-bar volume and found that the volume was similar during the day and night in all groups (two-way ANOVA, *p* = 0.4607, F_57_ = 0.5516) (Fig. 4E). However, the platform volume was larger during the night in Canton S than in the other mutants studied (two-way ANOVA, *p* < 0.0001, F_57_ = 16.65; *brp*^Δ170^: *p* < 0.001, t = 4.759; *brp*^Δ190^; *p* < 0.001, t = 6.260; *cry*^01^: *p* < 0.001, t = 5.109) (Fig. 4E). The platform was also larger at ZT1 in Canton S than in *brp*^Δ190^ (*p* < 0.01, t = 2.942) and *cry*^*01*^ (*p* < 0.01, t = 2.969). It indicates that the platform seems to be used during high activity of synapses and its build up to the regular size depends on CRY, BRP-170 and BRP-190 proteins, which influence the number of dense-core vesicles delivered to the platform during the night.

**Figure 5:**
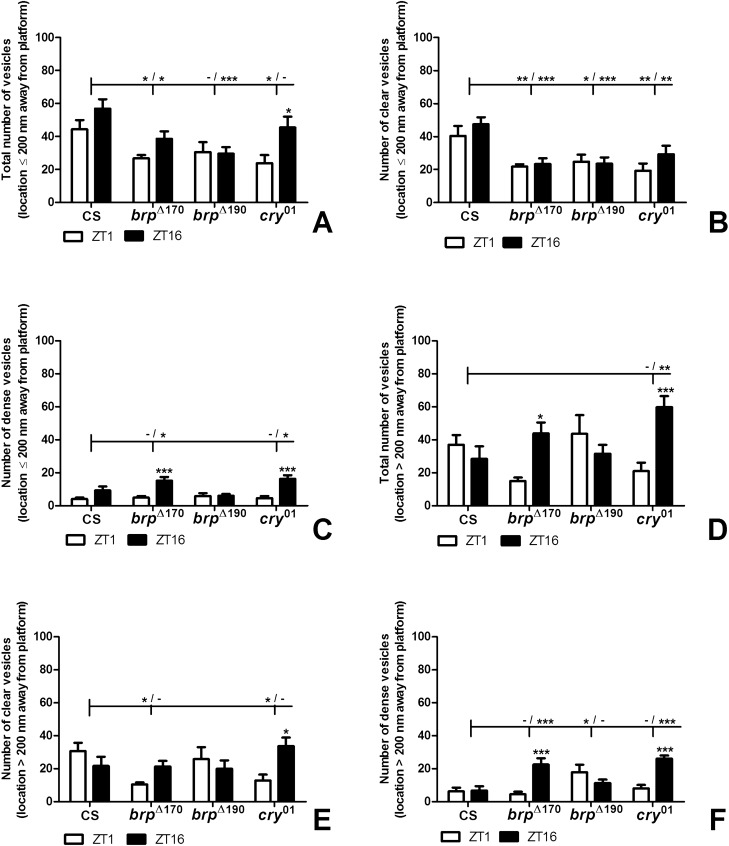
(**A**–**F**) Numbers of vesicles (total, clear and dense-core) depending on their distance from the T-bar: < 200 nm and > 200 nm. Asterisks indicate the following statistically significant differences: *p < 0.05, **p < 0.01, ***p < 0.001. Asterisks above bars show statistically significant differences between ZT1 and ZT16 within every group. Asterisks above horizontal lines show statistically significant differences between Canton S and mutants (*brp*^Δ170^**,** *brp*^Δ190^ and *cry*^01^), and minus means the lack of a statistically significant difference. Means ± SEM.

The number of all synaptic vesicles was also analysed in a short distance, 200 nm from the T-bar, and in a long distance, above 200 nm. The total number of vesicles in the short distance (Fig. 5A) followed the pattern observed for all synapses shown in Fig. 4A. The statistically significant day/night difference was observed only in the *cry*^01^ mutant (two-way ANOVA, *p* = 0.0029, F_57_ = 9.677; *p* < 0.05, t = 2.977) with more vesicles at ZT16 (91.58%). In Canton S, there were more synaptic vesicles than in *brp*^Δ170^ at ZT1 (66%) (two-way ANOVA, *p* = 0.0007, F_57_ = 6.518; *p* < 0.05, t = 2.413) and at ZT16 (47%) (*p* < 0.05, t = 2.572), and more vesicles were counted in Canton S than in *brp*^Δ190^ at ZT16 (92%) (*p* < 0.001, t = 3.839) and *cry*^01^ at ZT1 (87%) (*p* < 0.05, t = 2.824) (Fig. 5A). In the case of clear vesicles in the distance shorter than 200 nm, there were no statistically significant day/night differences in any group, but there was more vesicles in Canton S than in *brp*^Δ170^ at ZT1 (85%) (two-way ANOVA, *p* < 0.0001, F_57_ = 11.13; *p* < 0.01, t = 3.011) and ZT16 (103%) (*p* < 0.001, t = 4.05), in *brp*^Δ190^ at ZT1 (63%) (*p* < 0.05, t = 2.544) and ZT16 (102%) (*p* < 0.001, t = 4.03) and in *cry*^01^ at ZT1 (109%) (*p* < 0.01, t = 3.44) and ZT16 (63%) (*p* < 0.01, t = 3.067) (Fig. 5B). In contrast to all clear vesicles, vesicles located in the short distance were more reduced in the number in all mutants when compared with Canton S not only at ZT16 but also at ZT1.

The day/night difference was statistically significant, with more than three times vesicles at ZT16, for dense-core vesicles in the short distance in *brp*^Δ170^ (two-way ANOVA, *p* < 0.0001, F_57_ = 31.8; *p* < 0.001, t = 4.193) and *cry*^01^ (*p* < 0.001, t = 4.749). There was also more of this type of vesicle in *brp*^Δ170^ (63%) and *cry*^01^ (74%) than in Canton S at ZT16 (two-way ANOVA, *p* = 0.0063, F_57_ = 4.543; *p* < 0.05, t = 2.461 and *p* < 0.05, t = 2.877) (Fig. 5C).

For vesicles located in the distance longer than 200 nm, the day/night difference was detected in *brp*^Δ170^ (two-way ANOVA, *p* = 0.0182, F_57_ = 5.917; *p* < 0.05, t = 2.964) and *cry*^01^ (*p* < 0.001, t = 3.991) mutants with 2.9 times more vesicles at ZT16. In contrast to vesicles located in the short distance, those located above 200 nm were about 50% less numerous in Canton S than in *cry*^01^ at ZT16 (two-way ANOVA, *p* = 0.0004, F_57_ = 7.166; *p* < 0.01, t = 3.321) (Fig. 5D). The number of clear vesicles in the long distance from the T-bar was higher at ZT16 than at ZT1 only in *cry*^01^ (2.6 times) (two-way ANOVA, *p* = 0.0099, F_57_ = 4.157; *p* < 0.05, t = 3). These vesicles were similar in number between ZT1 and ZT16 in the other groups studied (Fig. 5E). There were, however, differences between Canton S and *brp*^Δ170^ at ZT1 (two-way ANOVA, *p* = 0.0099, F_57_ = 4.157; *p* < 0.05, t = 2.91) and *cry*^01^ at ZT1 (*p* < 0.05, t = 2.568), with more vesicles in Canton S than in *brp*^Δ170^ (2.9 times) and *cry*^01^ (2.4 times) (Fig. 5E). In the case of dense-core vesicles in the distance longer than 200 nm, the statistically significant difference between the day (ZT1) and night (ZT16) was found in *brp*^Δ170^ and *cry*^01^ mutants with 5 and 3.2 times, respectively more vesicles at ZT16 (Fig. 5F).

In contrast to clear synaptic vesicles, there were fewer dense-core vesicles in the distance above 200 nm in Canton S than in *brp*^Δ170^ at ZT16 (two-way ANOVA, *p* = 0.0028, F_57_ = 5.273; *p* < 0.001, t = 4.058), *brp*^Δ190^ at ZT1 (*p* < 0.05, t = 2.917) and *cry*^01^ at ZT16 (*p* < 0.001, t = 4.994) (Fig. 5F). The day/night differences in the number of vesicles were statistically significant for dense-core vesicles in *brp*^Δ170^ (two-way ANOVA, *p* = 0.0004, F_57_ = 14.39; *p* < 0.001, t = 4.515) and *cry*^01^ (*p* < 0.001, t = 4.516) with 5 and 3.2 times, respectively more vesicles at ZT16 than at ZT1 (Fig. 5F).

In general there were more clear vesicles than dense-core vesicles in the short distance from T-bars while dense-core vesicles were more numerous than clear ones in the longer distance, particularly at night in *brp*^Δ170^ and *cry*^01^.

### Numbers of tetrad synapses in Canton S and the ***cry***^***01***^ mutant

**Figure 6:**
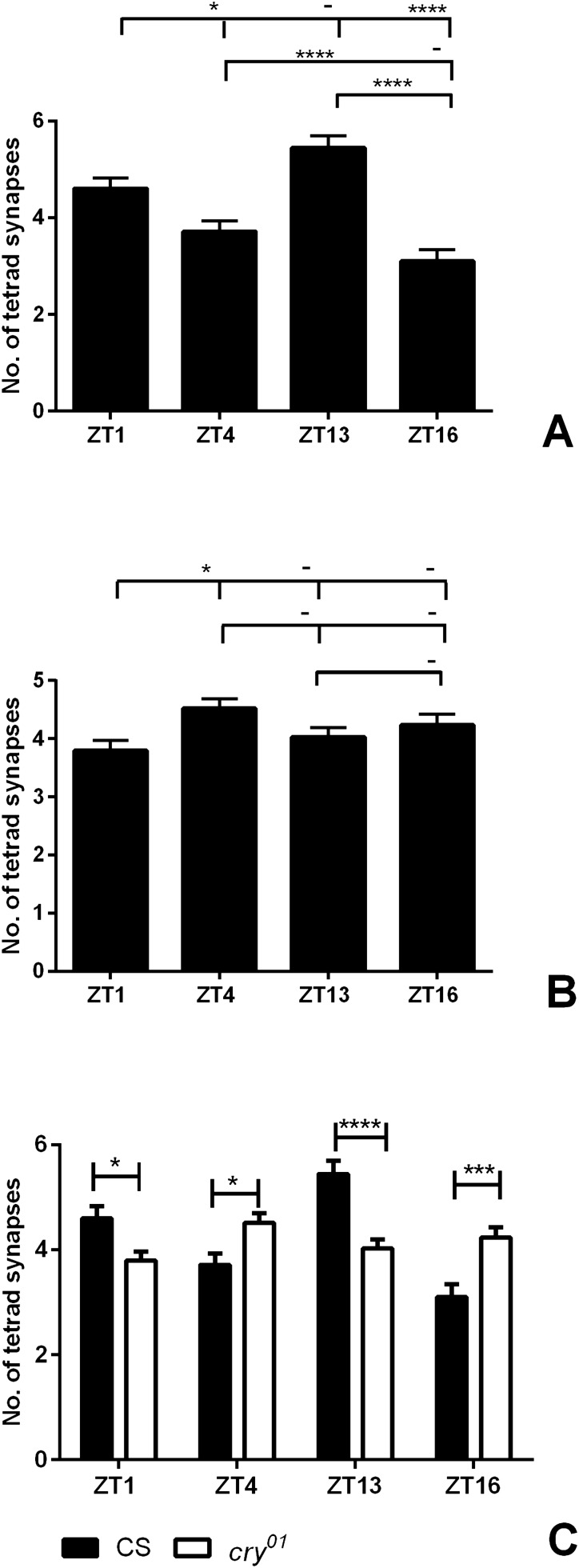
The daily rhythm in the numbers of tetrad synapses in the lamina of *D. melanogaster.* Daily changes of tetrad synapse number in wild-type Canton S (**A**) and the *cry*^*01*^ null mutant (**B**) examined at different time points. Statistically significant differences are marked with asterisks. (**C**) Comparisons between wild-type and mutant flies at every time point are shown separately. Statistically significant differences are marked with asterisks: *p ≤ 0.05; ***p ≤ 0.01; ****p ≤ 0.001. Mean ± SEM. N = 5 (CS), 7 (*cry*^*01*^).

The number of tetrad presynaptic profiles of wild-type flies showed daily changes with two peaks at ZT1 and ZT13 (Fig. 6A) (one-way ANOVA, *p* < 0.0001, F = 18.77, Tukey’s multiple comparisons test: ZT1 vs ZT4 *p* ≤ 0.05, ZT1 vs ZT16 *p* ≤ 0.001, ZT4 vs ZT13 *p* ≤ 0.001, ZT13 vs ZT16 *p* ≤ 0.001), which corresponds with the BRP expression pattern in the distal lamina, where tetrad synapses outnumber other synapse types^15^, and the number of tetrad synapses reported in our earlier study^14^. In *cry*^*01*^ mutants, this pattern changed, with more synapses in the middle of the day and in the middle of the night and fewer at the beginning of the day at ZT1 (one-way ANOVA, *p* = 0.0287, F = 3.044, Tukey’s multiple comparisons test: ZT1 vs ZT4 *p* ≤ 0.05) (Fig. 6B). The comparison between Canton S and *cry*^*01*^ at every time point studied showed that CRY affects the number of tetrad synapses and its daily oscillation. We observed fewer synapses at ZT1 (27%) and ZT13 (26%) and more at ZT4 (21.6%) and ZT16 (36.5) (two-way ANOVA, *p* < 0.0001, F = 16.99, Bonferroni’s multiple comparisons test: ZT1 *p* ≤ 0.05, t = 2.681, ZT4 *p* ≤ 0.05, t = 2.775, ZT13 *p* ≤ 0.001, t = 4.757, ZT16 *p* ≤ 0.01, t = 3.699) (Fig. 6C).

### T-bar size in the *cry*^*01*^ mutant

**Figure 7:**
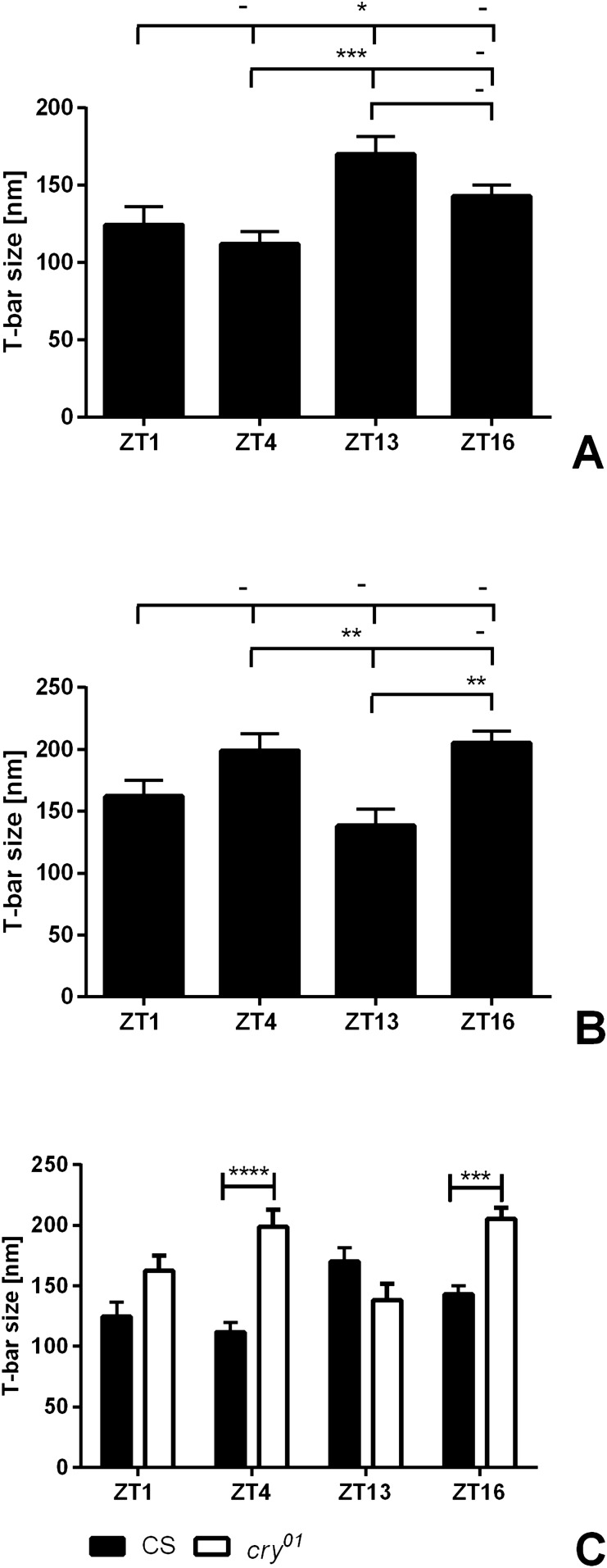
The daily rhythm in changes of the T-bar platform size of tetrad synapses in the lamina of *D. melanogaster*. Daily changes were observed in Canton S with the largest T-bar platforms at ZT13 (**A**). The pattern of daily changes was changed in *cry*^*01*^ mutants, and the smallest platforms were observed at ZT13 (**B**). Statistically significant differences are marked with asterisks. (**C**) Comparisons between wild-type and mutant flies at every time point are shown separately. Statistically significant differences are marked with asterisks. ***p ≤ 0.01; ****p ≤ 0.001. Mean ± SEM. N = 5 (CS), 7 (*cry*^*01*^).

T-bars of tetrad synapses in Canton S were significantly larger at ZT13 than at ZT1 and ZT4 (one-way ANOVA, *p* = 0.0006, F = 6.711, Tukey’s multiple comparisons test: ZT1 vs ZT13 *p* ≤ 0.05, ZT4 vs ZT13 *p* ≤ 0.01) (Fig. 7A), while in *cry*^*01*^, the T-bars were smaller at ZT13 than at ZT4 and ZT16 (one-way ANOVA, *p* = 0.0008, F = 6.568, Tukey’s multiple comparisons test: ZT4 vs ZT13 *p* ≤ 0.0005, ZT13 vs ZT16 *p* ≤ 0.005) (Fig. 7B). The T-bar structure was changed in *cry*^*01*^ relative to Canton S at ZT4 (larger by 77.7%) and ZT16 (larger by 43.5%) (two-way ANOVA, *p* < 0.0001, F = 10.7, Bonferroni’s multiple comparisons test: ZT4 *p* ≤ 0.001, t = 6.505, ZT16 *p* ≤ 0.01, t = 4.037) (Fig. 7C).

## Discussion

The obtained results confirmed our earlier studies that the presynaptic element (T-bar) of tetrad synapses is remodelled during the day and night and this rhythm, as we reported previously, is regulated by light and circadian clock^14,15^. In the present study, we found additionally that cyclic changes occur in the T-bar ultrastructure and its volume. Ultrastructural changes in T-bars and synaptic vesicles were possible because of using high resolution electron microscope tomography (EMT). In the present study we also found that the number of synaptic vesicles cycles during the day, but this rhythm is masked by light. This result indicates that in the case of tetrad synapses, which are sites of fast neurotransmission between photoreceptors and the first order interneurons, intense neurotransmission occurs during the morning peak of locomotor activity, when more synaptic vesicles are attached to the T-bar platform than during sleep (ZT16) and are transported from capitate projections located next to the T-bars. At ZT16, tetrad synapses are ready for neurotransmission, since the total number of synaptic vesicles near tetrad synapses is similar at ZT1 and ZT16, but it is in a standby mode with lower frequency of synapses and vesicles, which are not attached to the T-bar platform and are not delivered from glial cells, respectively. However, transmission can be activated and is efficient because synapses during sleep have larger volume, and synaptic vesicles can be transported to the T-bar platform, if necessary, in response to an unexpected light pulse. Cyclic remodelling of tetrad presynaptic sites depends on BRP, which must be delivered to T-bars and degraded after light exposure after binding to CRY. This fast remodelling of synapses affects the number of vesicles transported to the presynaptic element.

The present study was carried out only in light/dark (LD 12:12) conditions because in our earlier studies we found that the rhythm of the changes in BRP abundance in tetrad synapses of *D. melanogaster* is circadian. The rhythm is maintained in constant darkness (DD) and abolished in the *per*^*01*^ clock null mutant^15^. On the basis of these results, we assume that rhythmic changes during the 24 h cycle reported is this paper are also circadian; however, in DD, the morning peak in BRP is not present because it depends on light.

Electron microscope tomography (EMT) used in our study showed that synaptic vesicles are attached to the platform of the T-bar with filamentous proteins that are reduced in *brp*^Δ170^ and *brp*^Δ190^ mutants, which have fewer synaptic vesicles compared with wild-type flies. We also detected two types of vesicles, clear and dense-core. Dense-core synaptic vesicles were less numerous than clear ones. It is known that synaptic vesicles of tetrad synapses contain histamine^7^, while the content of dense-core vesicles is unknown. It is possible that they carry presynaptic proteins to the T-bar. The comparison of ultrastructure of tetrad synapses in the morning peak of activity (ZT1) and during sleep (ZT16) indicates that more vesicles are attached to the T-bar platform and to capitate projections at ZT1, but this pattern is not present in *brp*^Δ170^, *brp*^Δ190^ and *cry*^01^. This result confirmed our earlier study ^16,32^ that both high motor activity in the morning and light exposure increase activity of the visual system. In the morning, there is intense transport of synaptic vesicles to T-bars and delivery of histamine in clear vesicles from the epithelial glial cells through capitate projections. The evidence for a role of capitate projections in neurotransmitter recycling has already been reported^33^ and now we showed, using EMT, that vesicles are produced from capitate projections and directly delivered to T-bars. This intense transport is damaged in all mutants studied, suggesting that both BRP isoforms and CRY are needed for this process. In addition, in the *brp*^Δ190^ mutant lacking BRP-190, the T-bar structure is less dense than in the other strains studied. In our previous study, we found that there are approximately 50% fewer synapses in *brp*^Δ190^ than in Canton S and *brp*^Δ170 14^. In the study by Matkovic et al.^21^ it has been reported that BRP isoforms are important for the formation of T-bars in neuromuscular junctions, and in *brp* mutants T-bars are smaller than in controls. T-bar height was reduced in *brp*^*Δ190*^, whereas the widths of pedestal and platform were reduced in both mutants. They also decrease transmission since the active zone was smaller in both mutants and the number of synaptic vesicles was reduced. These ultrastructural changes are correlated with cell physiology since the amplitude of evoked excitatory junctional current was decreased in both mutants with a stronger effect in *brp*^*Δ190*^.

The obtained reconstructions of tetrad T-bars from TEM serial sections of the lamina showed that although there were fewer synapses during the night (ZT16), the volume of the T-bar was larger at that time than in the morning (ZT1), while the total number of synaptic vesicles was similar. In contrast, a day/night difference (ZT1 vs. ZT16) in the number of vesicles was observed in *brp*^Δ170^ and *cry*^01^. This suggests that the CRY protein and BRP-170 are responsible for an increase in the number of synaptic vesicles during the morning peak of activity. Since CRY co-localizes with BRP and is involved in BRP degradation in the morning^27^, CRY is probably also involved in the degradation of other proteins of synaptic vesicle organization in the morning since the number of vesicles in *cry*^01^ was low in the morning but high at night (ZT16). It seems that in *cry*^01^, synaptic vesicles are not delivered to the photoreceptor terminals from capitate projections and tethered to the cytomatrix in the morning. It is also interesting that the daily rhythm in the number of vesicles is not maintained in BRP mutants, which confirms our earlier study^14^, and the BRP N-terminus, which lacks *brp*^Δ190^, is necessary to maintain daily remodelling of the T-bar structure. Although both isoforms participate in building the cytomatrix^21^ their functions seem to be different in the course of the day. It is also possible that CRY is not only responsible for degradation of synaptic proteins but also as a protein, what is known, affecting the clock. In another cell types, in clock neurons l-LNvs, CRY, except interaction with TIM, is responsible for blue light response and firing of the l-LNvs^29^. In the lamina we found that in *cry*^01^ mutant the daily rhythm in synapse number and their remodelling was delayed in phase and the day/night difference in Canton S increased when peaks in the number of synapses were shift to ZT4 and ZT16. In result the difference between ZT1 and ZT16 was increased in *cry*^01^.

BRP is also responsible for rapid remodelling of the presynaptic active zone (AZ), and as reported in *Drosophila* NMJ, presynaptic homeostatic potentiation increases the number of BRP molecules and other AZ proteins, Unc13A and RBP, within minutes^34^.

When synaptic vesicles were counted at two different distances from the T-bar, to 200 nm and above 200 nm, there were differences between clear vesicles containing histamine and dense-core ones located in both areas. More clear vesicles were located near the T-bar and fewer above 200 nm. In the case of dense-core vesicles, their number was similar in both areas. This difference was not so striking in mutants in the case of vesicles located next to the platform, but in *brp*^Δ170^ and *cry*^01^, there were more dense-core vesicles at ZT16 in the distance above 200 nm than closer to the T-bar. This result indicates that BRP-170 and CRY are important for the distribution of clear synaptic vesicles next to the T-bar as well as dense-core vesicles located above 200 nm from the presynaptic element. It is possible that dense-core vesicles contain T-bar proteins, probably BRP. When transport along the actin cytoskeleton is disrupted, the number of tetrad synapses decreases^35^, and rapid AZ remodelling also fails^34^.

The abovementioned ultrastructural changes depend on the level of the presynaptic scaffolding protein BRP, which changes in abundance during the day and night^15^. These changes are controlled by the daily expression of CRY^27^, which seems to have many functions in photoreceptors in addition to being the circadian clock photoreceptor. In our earlier study, we found that CRY interacts with BRP but only during light exposure and leads to the degradation of BRP during the day/light phase of the 24 h cycle^27^. This seems to be responsible for the decrease in BRP level in the middle of the day after its peak at the beginning of the day. The lack of CRY in *cry*^01^ mutants changes the pattern of the tetrad presynaptic profile frequency during the day and the size of the T-bar. However, the rhythm is not completely abolished, which indicates that other proteins are also involved in the daily remodelling of tetrad synapses. Since CRY plays several functions in photoreceptors, changes in the number of tetrad synapse and T-bar size in *cry*^01^ may result from different processes and lack of interactions of CRY with TIM and BRP. CRY is a component of the molecular clock^36^ and interacts with TIM^37^, and this may affect daily changes in the number and size of T-bars. In addition, light-activated CRY binds BRP and targets it to degradation^27^. We showed previously that flies with constitutively active CRY have low BRP level. In turn, *cry*^01^ mutants show changes in the pattern of BRP expression, with higher BRP level during the day (ZT4), at the time when wild-type flies have minimum of BRP expression^27^. The pattern of BRP expression is similar to the pattern of daily changes in tetrad synapse number, so it is possible that the number of synapses or T-bar size is directly dependent on the amount of BRP which oscillates during the day. However, CRY in the retina photoreceptors binds also actin and is involved in the organization of phototransduction cascade of proteins in rhabdomeres^38^. This may be also involved in the regulation of T-bar structure. The differences in T-bar size of *cry*^*01*^ are shown at time when in Canton S CRY is active (during the day) or its level increases (ZT16). At the beginning of the night the level of CRY is very low, so there was no effect on T-bar structure and no difference between CS and *cry*^*01*^ was observed.

The epithelial glial cells are important for many processes during phototransduction and in recycling neurotransmitters and other compounds during the night. Glia take up histamine and metabolize it to carcinin, which is next delivered to the photoreceptor terminals, and capitate projections are involved in this process^39^. Activity of glial cells is also controlled by the circadian clock. During the night, glial cells seem to be more active than neurons to recycle neurotransmitters, and many proteins, including proteins of ion pumps, are found at higher concentrations at that time^40,41^. The high number of synaptic vesicles near the tetrad T-bar during the morning peak of activity in *Drosophila* seems to depend on capitate projections invaginating from the epithelial glia to the photoreceptor terminals in the lamina of *Drosophila.*

Although the presynaptic cytomatrix can be rapidly remodelled with transmission strength, it is also affected by motor and visual system activity, external factors, such as light in the case of the visual system, and the circadian clock, showing plasticity and correlation to changes in behaviour during the day/night cycle. As we showed in the present study synaptic plasticity and synapse remodelling during the day is a complex process which involves presynaptic proteins of the T-bar as well as two types of synaptic vesicles, clear and dense-core, and glial cells. We also found that fast degradation of proteins involved in transmission is as important as pre- and postsynaptic protein synthesis.

## Methods

**Table:** Key resources table

Reagent or resource	Source	Identifier
**Chemicals, peptides, and recombinant proteins**		
Glutaraldehyde, EM Grade 25%	Polysciences	01909-10
Paraformaldehyde EM grade	Polysciences	00380-250
Cacodylic acid sodium salt trihydrate	Polysciences	01131-100
Propylene oxide, EM grade	Polysciences	00236-1
Poly/Bed 812	Polysciences	08792-1
Sucrose	J.T.Baker	57-50-1
CaCl2	J.T.Baker	10035-04-8
**Experimental models: organisms/strains**		
*Drosophila melanogaster*: wild-type CantonS	Bloomington Drosophila Stock Center	BDSC #64349
*Drosophila melanogaster*: *cry*^*01*^	Dolezelova et al.^42^	N/A
*Drosophila melanogaster*: *brp*^*Δ190*^	Matkovic et al.^21^	N/A
*Drosophila melanogaster*: *brp*^*Δ170*^	Matkovic et al.^21^	N/A
**Software and algorithms**		
3D Studio Max	Discreet Logic, Montreal, Canada	
EM MENU 4 and ET Tools	Tietz Video & Image Processing Systems GmbH, Gauting, Germany	
IMOD ETomo	Kremer et al.^43^	https://bio3d.colorado.edu/imod/
GraphPad Prism 5.01	GraphPad Software Inc., USA	https://www.graphpad.com/scientific-software/prism/
ImageJ	Schneider et al.^44^	https://imagej.nih.gov/ij/

### Contact for reagent and resource sharing

Further information and requests for resources and reagents should be directed to and will be fulfilled by the Lead Contact, Elzbieta Pyza (elzbieta.pyza@uj.edu.pl).

### Experimental model and subject details

#### Fly strains

The following strains were used for experiments: wild-type Canton S, CRY null mutant (*cry*^*01*^)^42^, *brp*^*Δ190*^ (premature STOP codon at 261 aa of BRP-190)^21^, *brp*^*Δ170*^ (deletion of first exon of BRP-170)^21^. Both *brp* mutants were kindly provided by Dr. S. Sigrist (Free Univ. Berlin) and they were prepared using the 24,862 strain from Bloomington, which has w* background. They have been outcrossed into the wild-type background (CS) for six generations to remove the effect of w* background^21^. In case of *cry*^*01*^ mutant, this strain was constructed on *w*^*1118*^ background, by replacing *cry* region with the w^+mW.hs^ marker. In effect, these flies have *mini-white* expression which partially restores *w* mutation. Flies used in our study have red eyes which allows to use Canton S as controls.

Males, 7 days old, were used for experiments. Flies were kept in a light/dark regime (LD 12:12) (12 h of light and 12 h of darkness) under constant temperature 22 ± 1 °C and humidity of 60%. Heads were collected at selected time points: ZT1 (1 h after lights–on, the morning peak of locomotor activity), ZT4 (4 h after lights-on), ZT13 (1 h after lights-off) and ZT16 (4 h after lights–off, sleep) or only at ZT1 and ZT16. In LD 12:12, ZT0 means the beginning of the day/light and ZT12 means the beginning of the night/dark.

### Method details

#### Presynaptic element visualization by electron microscopy

Heads were fixed in 2% glutaraldehyde and 2.5% paraformaldehyde in a cacodyl buffer with CaCl_2_ for 1 h and postfixed for 1 h in 2% OsO_4_ in a veronal acetate buffer with CaCl_2_ and sucrose. Next, samples were dehydrated in an alcohol series and propylene oxide and embedded in Poly/Bed 812 (Polysciences) resin. Heads were collected at different times in LD 12:12 depending on the experiment. For analysis of the synapse number, Canton S and *cry*^01^ flies were fixed for TEM at ZT1, ZT4, ZT13, and ZT16, which were selected on the basis of our earlier studies^14^. For synapse ultrastructure and analyses of synaptic vesicles, flies were fixed at ZT1 and ZT16 only. We selected these two time points on the basis of our earlier studies, knowing that there is a statistically significant difference in the number of tetrad synaptic profiles between ZT1 and ZT16.

Ultrathin sections of the lamina were cut approximately 65 nm thick using a Leica EM UC7 ultracut and contrasted with uranyl acetate and lead citrate on grids. Images were taken with a Jeol JEM 2100 HT TEM.

To estimate the number of presynaptic elements in *cry*^01^ mutants and control (Canton S) flies, the presynaptic profiles of tetrad synapses were counted from 30 cartridges of 7–8 flies per time point. Each cartridge was photographed from three consecutive sections. The length of the presynaptic T–bar platforms was also measured at different time points. For measurements, only the longest cross sections of the platform were taken.

### 3D reconstruction and tomography of tetrad presynaptic profiles

Heads were fixed for TEM at ZT1 and ZT16, and then ultra-thin (70 nm) or thick (150 nm) sections of the lamina were cut for 3D tetrad presynaptic profile reconstructions and electron microscope tomography (EMT), respectively.

Four series of 10–12 constitutive cross sections (70 nm thick) of the lamina were cut from one fly (N = 4). They were viewed with TEM, and digital images of tetrad synaptic profiles were collected at magnification 60,000x. T-bars and synaptic vesicle 3D reconstructions were performed using 3D Studio Max software (Discreet Logic, Montreal, Canada). Two types of synaptic vesicles, clear and dense-core ones, were identified by EMT and counted from 10–12 serial sections at two distances from the T-bar: < 200 nm and > 200 nm. The number of serial sections was chosen to reconstruct the whole T-bars of tetrad synapses.

### Electron tomography

Sequential TEM imaging (120 images) was carried out at different angular orientations (between − 60° and + 60°) of thick (150 nm) sections using a Jeol 2100 HT at 200 kV. Images were collected at 60,000 × magnification with a TemCam-F416 camera, TVIPS (Tietz Video and Image Processing Systems) using EM MENU 4 and ET Tools software and a 4 K camera with 4096 × 4096 resolution. IMOD ETomo software was used for 3D reconstructions.

### Statistical analysis

To analyse the distribution of synaptic vesicles in tetrad presynaptic sites, two-way ANOVA followed by the Bonferroni test (GraphPad Prism 5.01 software, GraphPad Software Inc., USA) was used. The T-bar platform area and the number of vesicles were measured and counted, respectively, using NIH ImageJ software. To calculate the volume of the T-bar platform, the platform area was multiplied by the number of sections (thickness of one section was 0.07 µm) of the platform. The number of vesicles per 1 µm^3^ of the platform was calculated by dividing the total number of vesicles near this platform by its volume.

## Supplementary information

Supplementary Video 1.

Supplementary Video 2.

Supplementary Video 3.

Supplementary Video 4.

Supplementary Video 5.

Supplementary Video 6.

Supplementary Video 7.

Supplementary Video 8.

Supplementary Figures.

## Data Availability

Data are available on request by contacting Elzbieta Pyza (elzbieta.pyza@uj.edu.pl).
