# Supplementary material for: CRY-dependent plasticity of tetrad presynaptic sites in the visual system of Drosophila at the morning peak of activity and sleep
Source: Sci Rep. 2020 Oct 23;10:18161. doi: 10.1038/s41598-020-74442-w (PMC7585400; doi:10.1038/s41598-020-74442-w)
Supplement: Supplementary file 9 — Supplementary Figures. [file 41598_2020_74442_MOESM9_ESM.pdf]

CRY- dependent plasticity of tetrad presynaptic sites in the visual system of *Drosophila* at the morning peak of activity and sleep

Milena Damulewicz[1], Olga Woźnicka[1], Małgorzata Jasińska[2], and Elżbieta Pyza[1]

[1]Department of Cell Biology and Imaging, Institute of Zoology and Biomedical Research, Jagiellonian University, [2]Department of Histology, Jagiellonian University Medical College

Correspondence: elzbieta.pyza@uj.edu.pl

Supplementary Materials

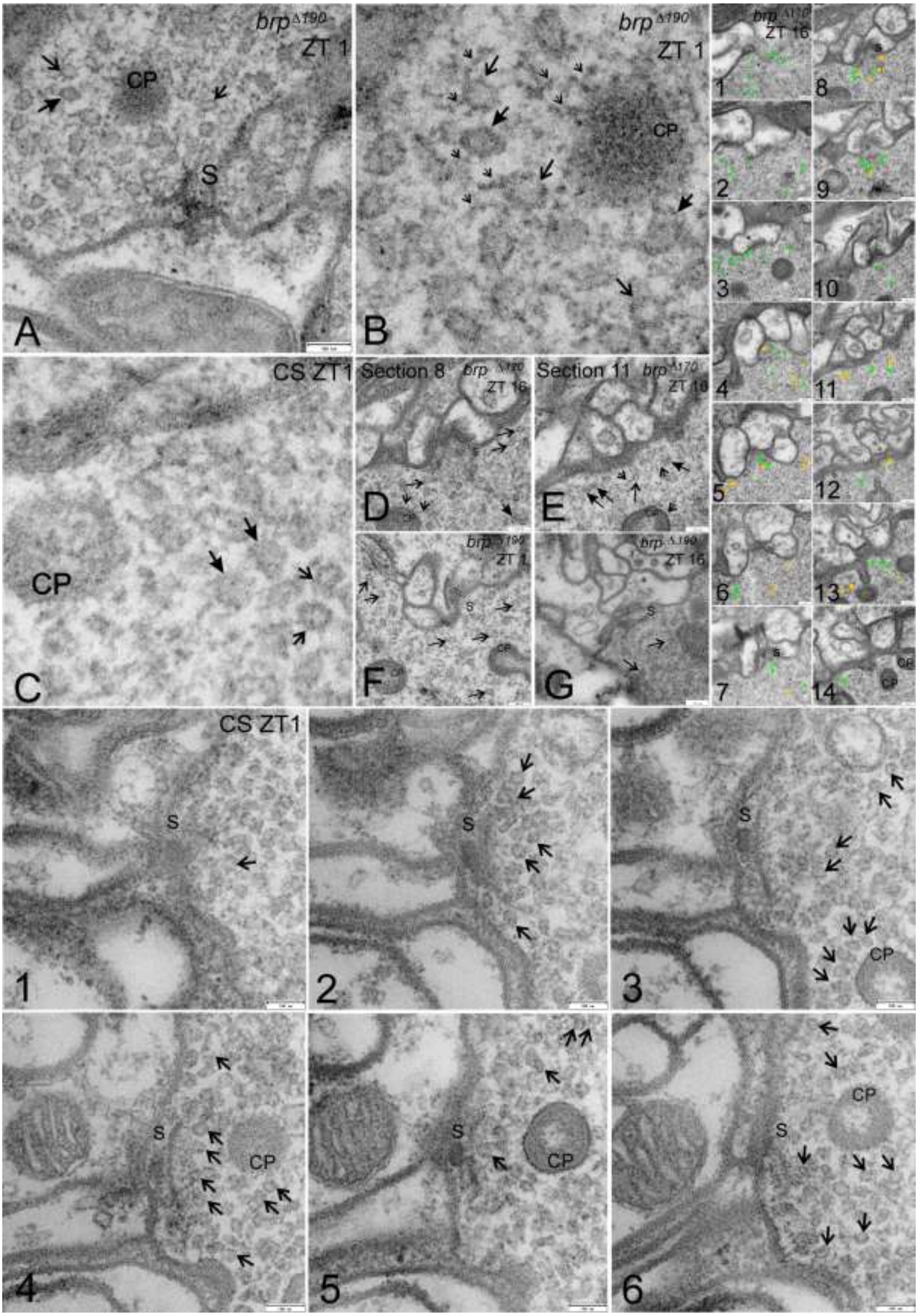

Figure S1. Examples of synaptic vesicles in Canton S and the *brp* mutants.

A, B– Dense-core and clear vesicles in the *brp*$^{\Delta 190}$ mutant and Canton S (C) at ZT1; D, E – vesicles in the *brp*$^{\Delta 170}$ mutant at ZT16 and in *brp*$^{\Delta 190}$ at ZT1 and ZT16 (F, G). 1-14 – consecutive sections of tetrad presynaptic element. Clear vesicles – short arrows, dense-core vesicles – black arrows, filaments – small arrows.

Bottom panel (1-6) shows serial sections of the T-bar in Canton S at ZT1. Clear synaptic vesicles are located next to the T-bar and capitate projections (short arrows). S – synapses, CP – capitate projections. Scale bar: 100 nm Magnifications: x60.000.

A

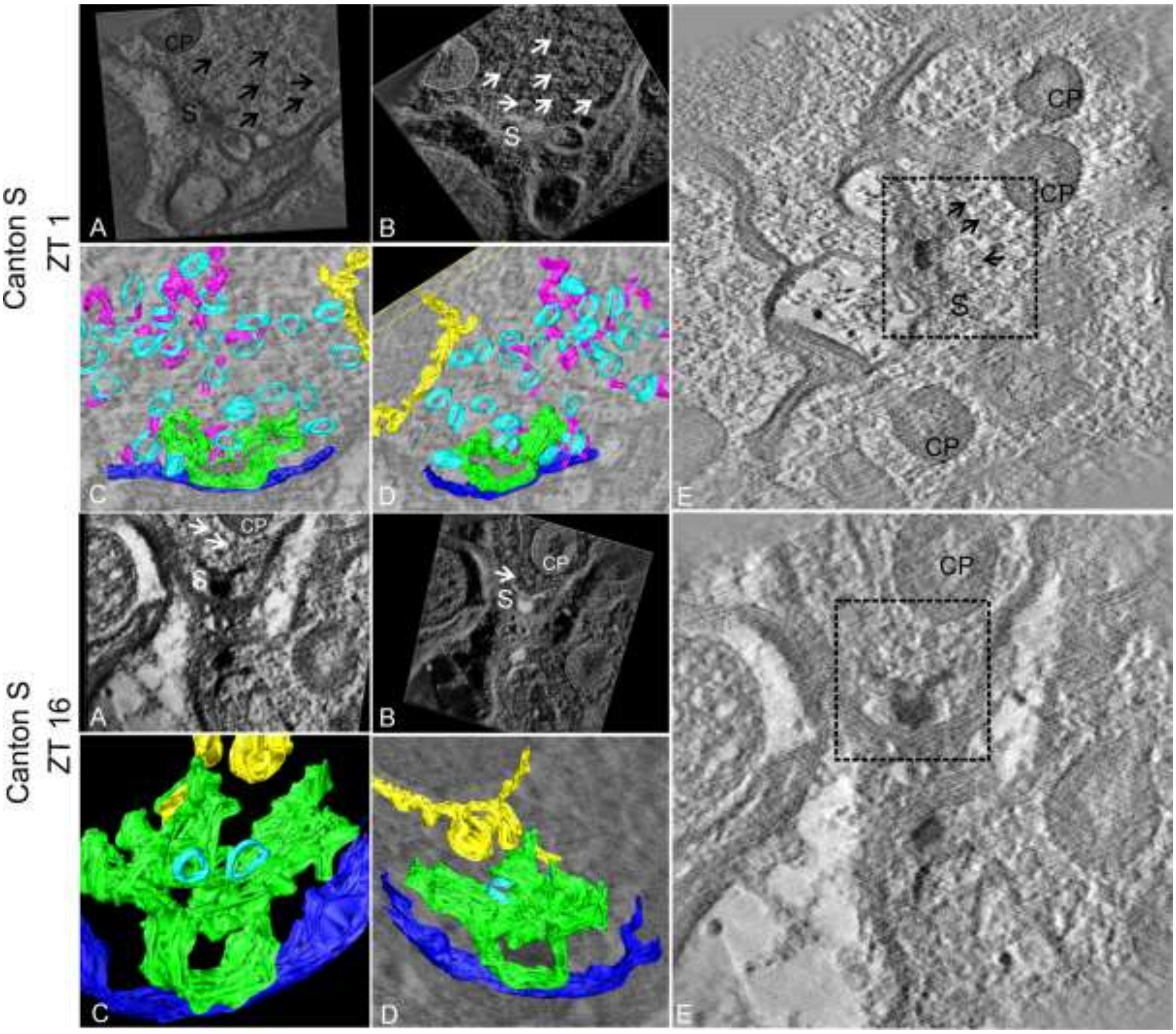

B

brp $^{\Delta 170}$
ZT1

brp $^{\Delta 170}$
ZT16

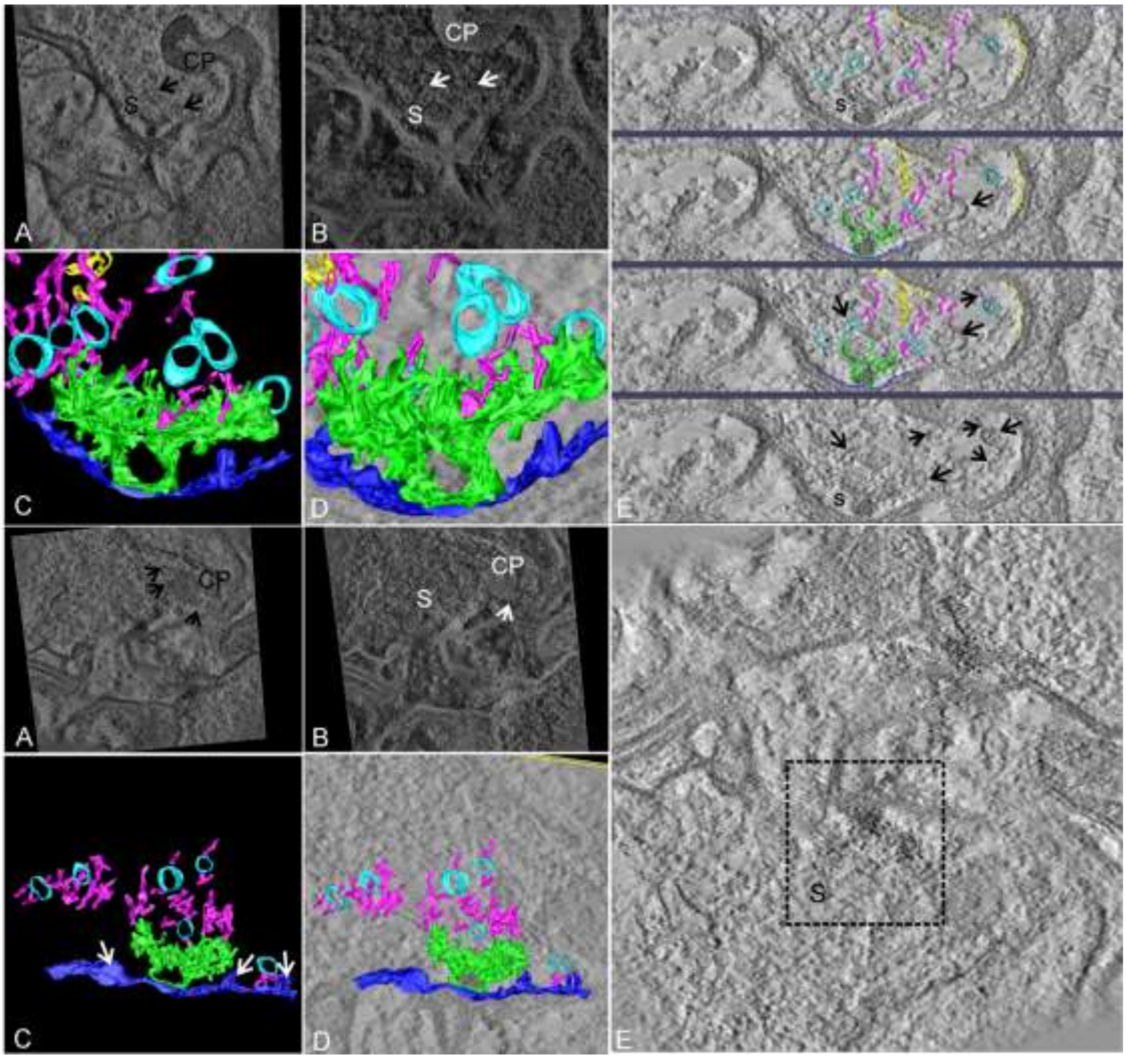

C

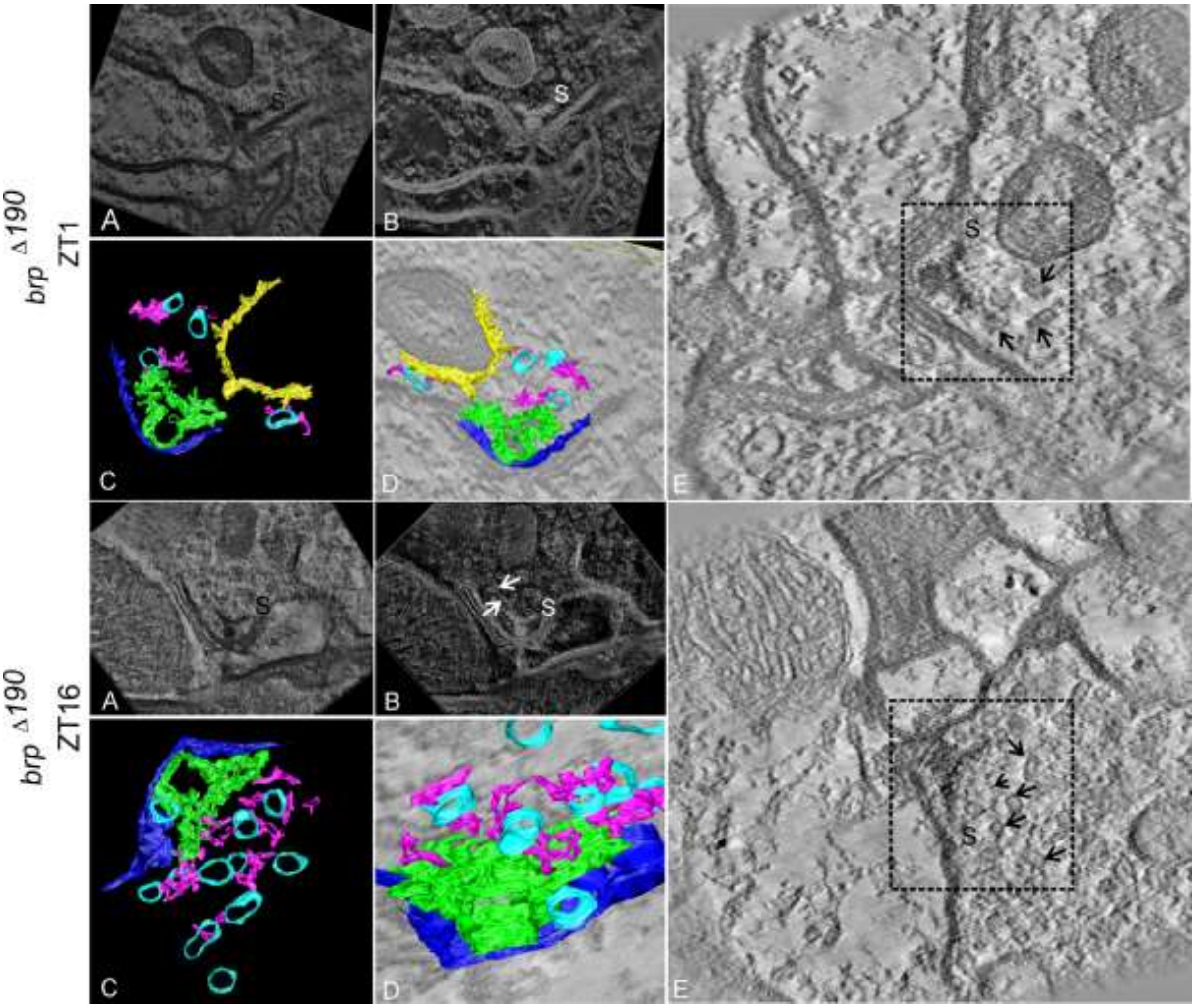

D

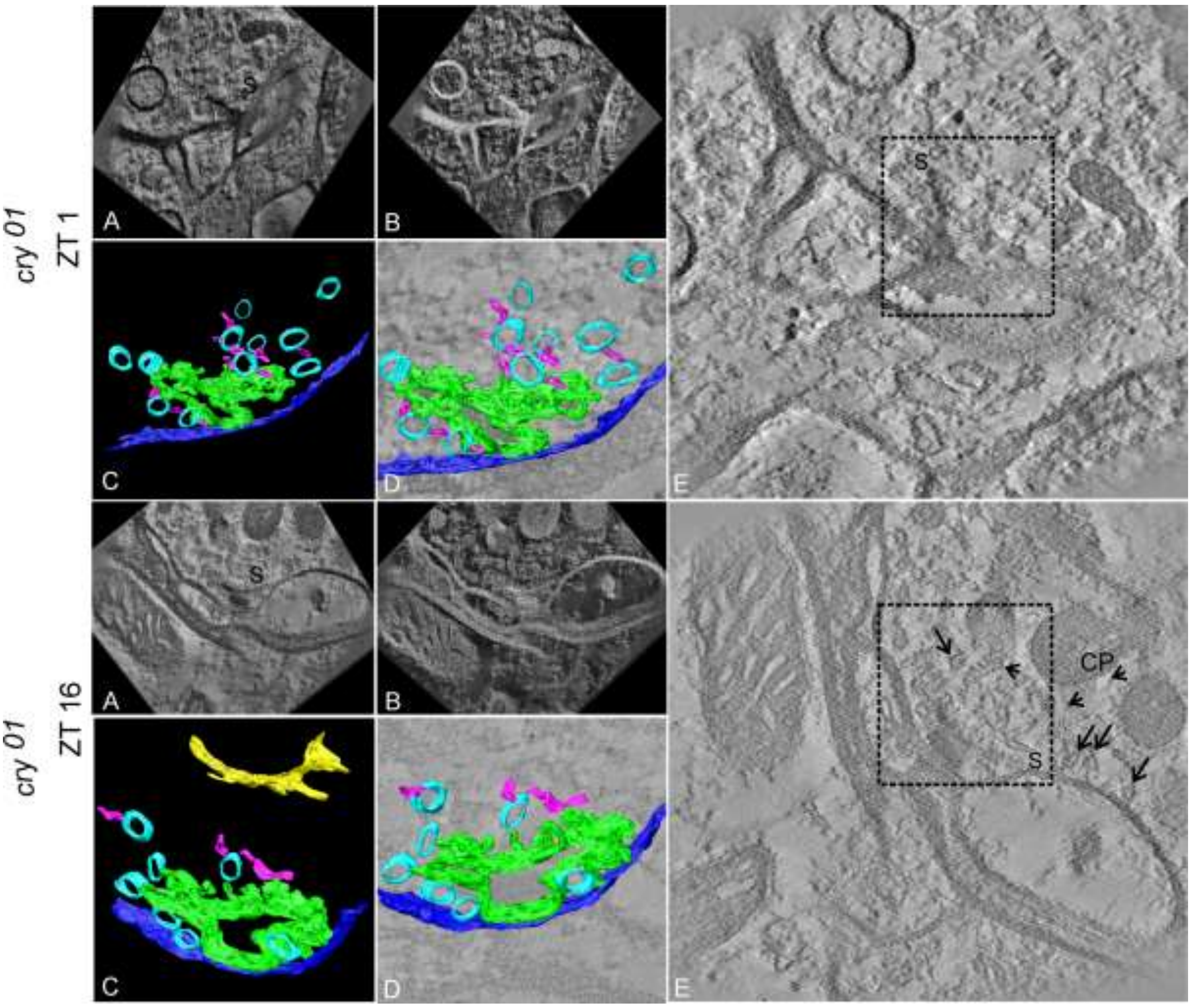

Figure S2A-D. Examples of synapse and synaptic vesicles reconstructions using eTOMO (E), UCSF Chimera (A,B), 3d Imod software (C, D).

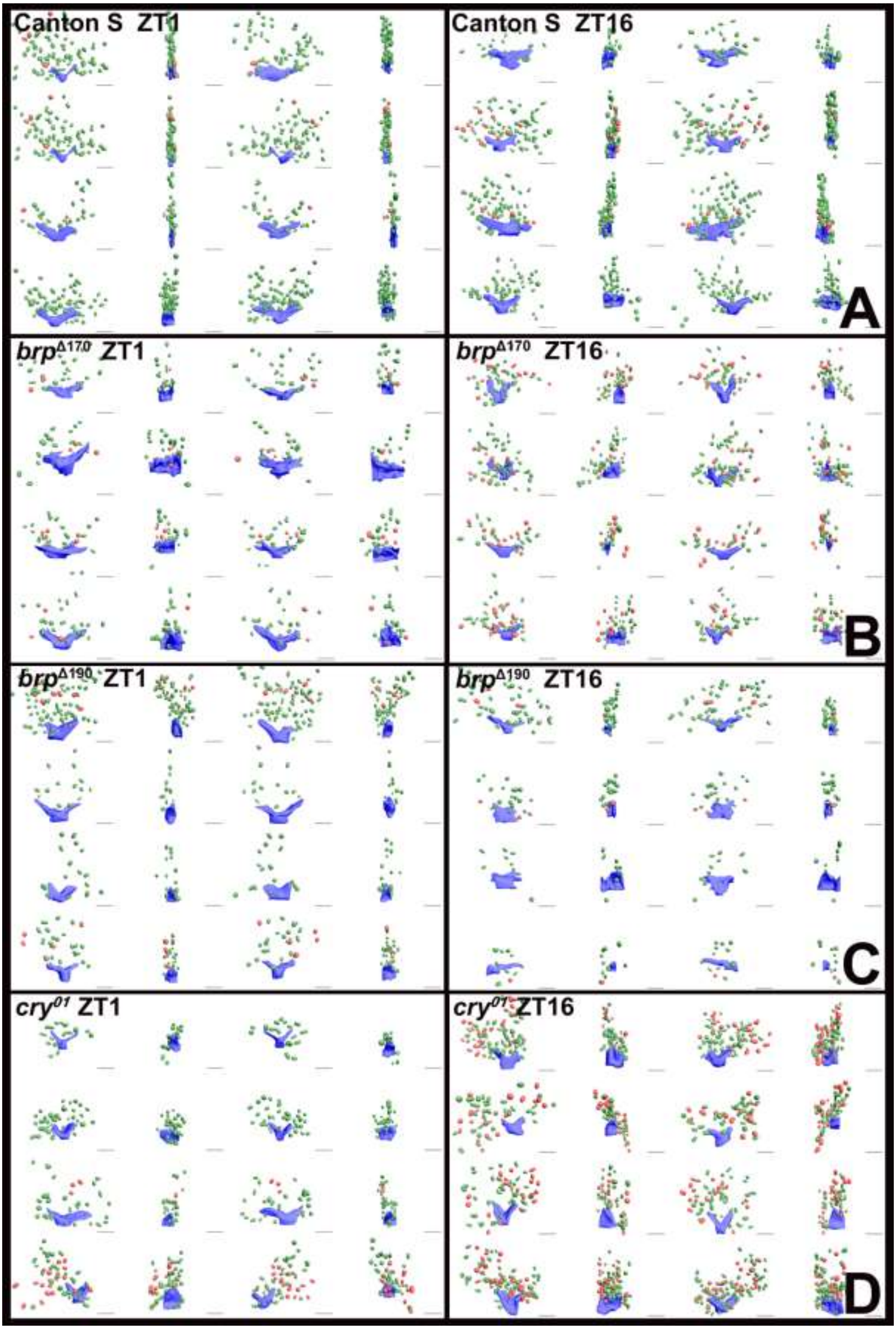

Canton S ZT1
Canton S ZT16
A
brpΔ170 ZT1
brpΔ170 ZT16
B
brpΔ190 ZT1
brpΔ190 ZT16
C
cry01 ZT1
cry01 ZT16
D

Figure S3 Four side views of TEM 3D reconstructions from serial sections of T-bars (blue) with synaptic vesicles (clear – green, dense-core – red) in Canton S and mutants at ZT1 and ZT16. Scale bar = 100 nm.

Figure S4 Animation of T-bar and vesicles in Canton S at ZT1 (CantonS_ZT1_an), ZT16 (CantonS_ZT16_an).

Figure S5 Animation of T-bar and vesicles in *brp*$^{\Delta 170}$ **at ZT1 (BRP170_ZT1), ZT16 (BRP170_ZT16_an).**

Figure S6 Animation of T-bar and vesicles in *brp*$^{\Delta 190}$ **at ZT1 (BRP190_ZT1_an), ZT16 (BRP190_ZT16_an).**

Figure S7 Animation of T-bar and vesicles in ***cry***$^{\mathbf{01}}$ **at ZT1 (Cry0_ZT1_an), ZT16 (Cry0_ZT16-an).**
